# Triglycerides as a mediator in the hyperuricemia-diabetes link: insights from a hypertensive Chinese population

**DOI:** 10.3389/fendo.2025.1645766

**Published:** 2025-09-11

**Authors:** Dan Lu, Xiaowen Ou, Tingjun Wang, Guoyan Xu

**Affiliations:** ^1^ Department of General Medicine, The First Affiliated Hospital of Fujian Medical University, Fuzhou, Fujian, China; ^2^ Department of General Medicine, National Regional Medical Center, Binhai Campus of the First Affiliated Hospital, Fujian Medical University, Fuzhou, Fujian, China

**Keywords:** hyperuricemia, diabetes mellitus, hypertension, triglyceride, mediation analysis

## Abstract

**Background:**

This study investigates whether triglycerides mediate the association between hyperuricemia and Type 2 Diabetes Mellitus (T2DM) in a hypertensive Chinese population. By comparing individuals with and without diabetes and applying generalized structural equation modeling (GSEM), we aimed to clarify the indirect effects of hyperuricemia via triglyceride pathways.

**Methods:**

A total of 274 hypertensive diabetic patients were assessed for clinical and biochemical profiles. The study included clinical categorization of DM status, demographic analysis, generalized structural equation modeling (GSEM) for mediation analysis, and regression modeling to identify diabetes risk factors.

**Results:**

Diabetic individuals exhibited significantly higher triglyceride levels (P=0.005). Age ≥65 years was a notable demographic risk factor compared to those aged 32–49 years (OR=6.35, 95% CI: 1.26–31.97). Occasional smoking also increased DM risk (OR=3.92, 95% CI: 1.00–15.35), while alcohol consumption showed no significant association. Hyperuricemia was positively associated with elevated triglyceride levels (coefficient = 0.67, P=0.01), which, in turn, significantly increased DM risk (coefficient = 1.29, P < 0.001). Although the direct effect of hyperuricemia on DM was not statistically significant (coefficient = -0.61, P=0.10), the indirect effect mediated by triglycerides was substantial (coefficient = 0.87, P=0.04). BMI categorization significantly influenced both hyperuricemia and triglyceride levels, with the highest BMI category (≥27) exhibiting the greatest prevalence (60.26% and 38.46%, respectively). However, the direct association between BMI and DM was not statistically significant (P=0.407), suggesting the involvement of mediating factors.

**Conclusion:**

Triglycerides play a key mediating role in the relationship between hyperuricemia and Type 2 DM among hypertensive patients. BMI is significantly associated with hyperuricemia and triglyceride levels, although not directly with DM. These findings emphasize the need for targeted interventions focused on lipid regulation, weight control, and lifestyle modifications to prevent diabetes progression in this high-risk population.

## 1 Introduction

The complex interplay between metabolic disorders such as hyperuricemia and Type 2 Diabetes Mellitus (T2DM) in hypertensive populations poses significant clinical challenges. Hyperuricemia, characterized by elevated serum uric acid levels, has been increasingly recognized as a potential risk factor for the development of T2DM, underscoring the need for a deeper understanding of its pathophysiological impact on glucose metabolism and insulin sensitivity (1, 2). Emerging evidence suggests that hyperuricemia may contribute to insulin resistance, thereby accelerating the progression toward diabetes (3, 4).

Additionally, elevated triglyceride levels, which often co-occur with hyperuricemia, further aggravate insulin resistance a central mechanism in the pathogenesis of T2DM. These lipid abnormalities are particularly prevalent among hypertensive individuals, who are already predisposed to metabolic dysregulation (5). Effective management of triglyceride levels in this high-risk group has been shown not only to aid in glycemic control but also to reduce cardiovascular risk, thereby offering dual therapeutic benefits (6).

Building on these insights, extensive epidemiological studies across multiple countries—including Japan, Finland, the United States, the Middle East, Spain, China, and Taiwan—have consistently demonstrated a strong association between elevated serum triglyceride levels and an increased risk of developing hypertension, with follow-up periods ranging from 2.3 to 10.8 years (7–16). These findings reinforce the role of triglycerides as key biomarkers and potential therapeutic targets in the prevention and management of hypertension, particularly among patients at elevated risk for T2DM, highlighting the importance of comprehensive lipid management strategies in this population.

The complex, bidirectional relationship between hypertension and T2DM has been further clarified by recent research, including a prominent Mendelian randomization (MR) analysis. This study, involving over 300,000 individuals, suggested that T2DM may causally contribute to the development of hypertension, whereas the reverse relationship appears less definitive, indicating that diabetes may act as a driver of hypertension rather than the outcome (17). These genetic findings support the rationale for targeted interventions aimed at reducing hyperglycemia as a strategy to mitigate hypertension risk, rather than relying solely on hypertension control to prevent diabetes.

Globally, hypertension and T2DM are among the leading cardiovascular risk factors, contributing significantly to morbidity and mortality worldwide (18). The widespread prevalence and profound health impact of these conditions underscore the urgent need for more effective management and prevention strategies. The World Health Organization has identified both diseases as public health priorities due to their substantial role in the growing global burden of non-communicable diseases (19, 20) (21). Focusing on hypertensive individuals is clinically relevant because they represent a metabolically high-risk subgroup in which lipid abnormalities and insulin resistance are more prevalent. Studying this population provides valuable insights into disease progression and intervention strategies within a group already vulnerable to metabolic disorders.

This study aims to determine whether triglycerides mediate the association between hyperuricemia and Type 2 Diabetes Mellitus (T2DM) within a hypertensive population. Using a population-based cohort stratified by diabetes status, we applied generalized structural equation modeling (GSEM) to assess both direct and indirect effects. Although the direct association between hyperuricemia and T2DM was not statistically significant, a significant indirect effect was observed through elevated triglyceride levels. This finding aligns with previous population-based studies that support a mechanistic interplay among hyperuricemia, hypertriglyceridemia, and T2DM development, further highlighting the importance of addressing lipid metabolism in diabetes prevention strategies.

Also, this study investigates the mediating role of triglycerides in the association between hyperuricemia and Type 2 Diabetes Mellitus (T2DM) among hypertensive individuals—a novel contribution to the literature on metabolic disorders. While previous research has established independent associations between uric acid, triglycerides, and diabetes, few studies have evaluated whether triglycerides function as a mechanistic bridge linking hyperuricemia to T2DM. This question is especially relevant in hypertensive populations, who often exhibit metabolic clustering yet remain underrepresented in mechanistic pathway analyses. By applying generalized structural equation modeling (GSEM), our study uncovers an indirect pathway through which hyperuricemia contributes to diabetes risk via elevated triglyceride levels despite a nonsignificant direct effect. This novel insight suggests that targeting triglyceride metabolism may offer greater clinical benefit than focusing on uric acid alone and provides a new lens for understanding metabolic dysregulation in hypertensive patients.

## 2 Methods

This retrospective cohort study included participants aged 18 years and older, selected from a comprehensive health database in China. The study population consisted of individuals diagnosed with hypertension, based on the diagnostic criteria outlined in the Chinese Guidelines for the Prevention and Treatment of Hypertension (2018 Revision). Eligibility criteria required that participants had no prior history of antihypertensive medication use before diagnosis and had documented systolic blood pressure (SBP) ≥140 mmHg and/or diastolic blood pressure (DBP) ≥90 mmHg on at least three separate occasions. Additionally, individuals with a history of hypertension who were undergoing antihypertensive treatment and maintaining controlled blood pressure levels (<140/90 mmHg) were also included.

### 2.1 Exclusion criteria

Participants were excluded if they had a diagnosis of other cardiovascular conditions, including but not limited to stroke, congestive heart failure, coronary artery disease, myocardial infarction, valvular heart disease, or the presence of a pacemaker. Additional exclusion criteria included severe hepatic or renal dysfunction, cognitive or communication impairments, thyroid disorders, malignant tumors, chronic obstructive pulmonary disease, or any history of infection or surgery within the preceding three months. Individuals with active infections, inflammatory diseases, secondary hypertension, hematological or autoimmune disorders were also excluded. Pregnant or lactating women, those currently menstruating, and individuals with a history of alcohol abuse or drug misuse were not considered for inclusion.

The rationale for selecting a hypertensive population stems from the high coexistence rate of hypertension with metabolic disorders such as dyslipidemia and diabetes. Targeting this subgroup allows for more focused analysis of interrelated metabolic pathways and has direct implications for clinical risk stratification and intervention.

Diagnostic criteria were as follows: Hyperuricemia was defined as a serum uric acid (SUA) level >420 µmol/L (7.0 mg/dL) for men and >360 µmol/L (6.0 mg/dL) for women, consistent with commonly used clinical thresholds. Type 2 Diabetes Mellitus (T2DM) was diagnosed based on the American Diabetes Association (ADA) criteria: fasting plasma glucose ≥7.0 mmol/L (126 mg/dL), HbA1c ≥6.5%, or documented clinical diagnosis of diabetes in medical records.

### 2.2 Analytical approach

Generalized structural equation modeling (GSEM) was employed to examine the mediating role of triglycerides in the association between hyperuricemia and Type 2 Diabetes Mellitus among hypertensive patients. We explicitly adjusted for a-priori selected covariates, including age, sex, body mass index (BMI), smoking, and alcohol use, based on their clinical relevance and potential confounding effect in a hypertensive cohort. In sensitivity analyses, we further adjusted for renal function (serum creatinine) and medication use (antihypertensive, lipid-lowering, and urate-lowering therapies) to assess model robustness. A p-value of less than 0.05 was considered statistically significant.

We used generalized structural equation modeling (GSEM) to test whether triglycerides (TG) mediate the association between hyperuricemia (HUA) and Type 2 diabetes mellitus (T2DM). The model contained three paths: (i) *HUA → TG* (Gaussian/identity link), (ii) *TG → T2DM* (logit link), and (iii) *HUA → T2DM* (logit link).

A parsimonious, a-priori set of confounders was included on the HUA→TG and TG→T2DM paths: age, sex, BMI, smoking, alcohol use. Given clinical relevance in a hypertensive cohort and potential confounding, we additionally adjusted for renal function (serum creatinine) and medication use (indicator variables for antihypertensive therapy, lipid-lowering therapy, and urate-lowering therapy) in sensitivity analyses.

Estimation and inference: direct (*c′*), indirect (*a×b*), and total effects were estimated; bias-corrected bootstrapped CIs (5,000 resamples) were computed for the indirect effect. Because the direct and indirect effects point in opposite directions, we do not report “percent mediated”, which is not interpretable under inconsistent mediation/suppression. Instead, we report path-specific effects with CIs.

### 2.3 Quality control

Strict quality control protocols were implemented to ensure data accuracy and reliability. These included data validation procedures and the calibration of diagnostic instruments in accordance with current clinical guidelines (22).

### 2.4 Ethical considerations

This study was approved by Medical Research and Clinical Technology application Branch of the Ethics Committee of the First Affiliated Hospital of Fujian Medical University ([2015]084-2). All participants provided informed consent prior to inclusion in the study. Data was managed in strict accordance with confidentiality protocols to ensure the protection of participant privacy and compliance with ethical research standards.

### 2.5 Statistical analysis

Descriptive statistics were used to summarize the clinical, biochemical, and demographic characteristics of the study population. Continuous variables were presented as means with standard deviations (SD), and categorical variables were expressed as frequencies and percentages. Group comparisons between hypertensive patients with and without Type 2 Diabetes Mellitus (T2DM) were conducted using independent sample *t*-tests for continuous variables and chi-square tests for categorical variables. To identify significant demographic and behavioral risk factors associated with T2DM among hypertensive individuals, multivariate logistic regression analysis was performed. Adjusted odds ratios (ORs) with 95% confidence intervals (CIs) were calculated to assess the strength of associations for factors including age, smoking status, and alcohol consumption. A forest plot was constructed to visualize the regression results and the effect sizes of each variable.

Generalized structural equation modeling (GSEM) was employed to explore the mediating effect of triglyceride levels in the relationship between hyperuricemia and T2DM. The model estimated both direct and indirect effects, with statistical significance assessed using corresponding path coefficients and *p*-values. The mediation effect was quantified by calculating the proportion of the total effect explained by the triglyceride pathway. All statistical analyses were performed using R version 4.5.1, with a two-tailed *p*-value < 0.05 considered statistically significant.

## 3 Results

### 3.1 Clinical and biochemical profiles of hypertensive patients

The clinical and biochemical characteristics of the hypertensive cohort were stratified based on the presence or absence of Type 2 Diabetes Mellitus (T2DM). Patients were categorized into diabetic (DM) and non-diabetic (non-DM) groups to facilitate comparison. Variables assessed included demographic parameters (such as age and sex), lifestyle factors (smoking and alcohol consumption), body mass index (BMI), and key biochemical indicators including serum triglycerides, uric acid, and fasting glucose levels. Significant differences were observed between the two groups in several metabolic markers, particularly triglyceride levels, which were elevated among diabetic individuals. A detailed summary of these findings is provided in **Table 1**.

**Table 1:** Clinical index of participants by type-2 diabetes mellitus (DM) groups.

Clinic index	Unit of variables	N	Mean (SD)	Median (IDR)	Min	Max	P25-P75	P
Liver								
ALT U/L	None-DM	229	25.78 (13.18)	23 (12)	9	89	17-29	0.29
	DM	43	27.77 (14.05)	25 (15)	10	72	18-33	
	Overall	272	26.10 (13.31)	24 (13)	9	89	17-30	
AST U/L	None-DM	230	22.01(6.13)	21 (6)	6	54	19-25	0.41
	DM	43	21.28 (6.10)	20 (8)	12	39	17-25	
	Overall	273	21.90 (6.13)	21 (7)	6	54	18-25	
TBIL	None-HBP	230	13.27 (5.21)	12.5 (6.1)	4.1	43	9.4-15.5	0.75
	DM	43	13.05 (4.86)	11.5 (5.8)	5	26.4	9.8-15.6	
	Overall	273	13.24 (5.15)	12.3 (5.9)	4.1	43	9.6-15.5	
ALB	None-DM	229	42.98 (3.03)	43 (4.2)	35.4	52	40.8-45	0.68
	DM	43	42.78 (2.78)	42 (4.3)	36.5	47.7	40.7-45	
	Overall	272	42.95 (2.99)	42.85 (4.25)	35.4	52	40.75-45	
Kidney								
BUN mmol/L	None-DM	230	5.41 (1.31)	5.33 (1.53)	2.67	10.21	4.52-6.05	0.15
	DM	43	5.61 (1.16)	5.65 (1.51)	2.38	9.65	4.77-6.28	
	Overall	273	5.45 (1.29)	5.36 (1.50)	2.38	10.21	4.59-6.09	
Cr µmol/L	None-DM	230	67.30 (15.45)	65.9 (20.9)	38.3	133.4	55.5-76.4	0.18
	DM	43	64.22 (15.98)	61.3 (18.9)	37.4	108.4	53.9-72.8	
	Overall	273	66.82 (15.54)	64.8 (19.8)	37.4	133.4	55.4-75.2	
UA	None-DM	233	399.43 (86.51)	395.5 (117.4)	219.7	806.4	331.9-449.3	0.10
	DM	43	374.16 (83.27)	361.6 (106.5)	195.4	603.0	322.7-429.2	
	Overall	276	395.49 (86.35)	394.4 (114.9)	195.4	806.4	331.9-446.7	
Blood								
Hb	None-DM	224	141.18 (13.03)	142 (17.5)	102	169	132.5-150.0	0.72
	DM	41	142.0 (14.93)	141 (19.0)	116	184	132.0-151.0	
	Overall	265	141.31 (13.32)	142 (18.0)	102	184	132.0-150.0	
RBC	None-DM	224	4.64 (0.46)	4.66(0.60)	3.22	6.19	4.32-4.92	0.24
	DM	41	4.73 (0.51)	4.59 (0.64)	3.90	5.98	4.37-5.01	
	Overall	265	4.65 (0.47)	4.65 (0.60)	3.22	6.19	4.33-4.93	
WBC	None-DM	224	6.02 (1.45)	5.89 (1.74)	3.06	12.16	5.03-6.76	0.06
	DM	41	6.47(1.53)	6.48 (1.83)	3.97	11.18	5.48-7.31	
	Overall	265	6.09 (1.47)	5.97(1.75)	3.06	12.16	5.07-6.82	
PLT	None-HBP	223	243.77 (58.12)	242 (74.0)	122	465	204.0-278.0	0.67
	DM	40	240.03 (51.46)	237 (65.5)	115	361	201.5-267.0	
	Overall	263	243.20 (57.08)	241 (71)	115	465	204-275	
NEUT	None-DM	223	3.38 (0.99)	3.28 (1.28)	1.11	7.45	2.68-3.96	0.19
	DM	41	3.65 (1.13)	3.55 (1.49)	2.00	7.01	2.80-4.29	
	Overall	264	3.4 (1.0)	3.35 (1.29)	1.11	7.45	2.69-3.98	
LYM	None-DM	223	1.95 (0.59)	1.83 (0.74)	0.78	4.83	1.50-2.24	0.11
	DM	41	2.07 (0.52)	1.95 (0.74)	1.25	3.15	1.67-2.41	
	Overall	264	1.96 (0.58)	1.86 (0.76)	0.78	4.83	1.53-2.28	
MONO	None-DM	223	0.36 (0.11)	0.34 (0.13)	0.16	0.77	0.28-0.41	0.06
	HBP	41	0.39 (0.11)	0.37 (0.18)	0.19	0.63	0.30-0.48	
	Overall	264	0.36 (0.11)	0.34 (0.14)	0.16	0.77	0.28-0.42	
RDW	None-DM	218	13.72 (1.57)	13.6 (1.4)	11.7	25.5	12.8-14.2	0.95
	DM	39	13.54 (0.81)	13.6 (1.4)	12.1	14.9	12.8-14.2	
	Overall	257	13.70 (1.48)	13.6 (1.4)	11.7	25.5	12.8-14.2	
Lipid								
TC	None-DM	230	4.55 (0.99)	4.48 (1.48)	2.45	7.28	3.83-5.31	0.84
	DM	43	4.51(0.96)	4.47 (1.32)	2.18	6.91	3.79-5.11	
	Overall	273	4.54 (0.98)	4.47 (1.40)	2.18	7.28	3.83-5.23	
TG (**) mmol/L	None-DM	230	1.77 (1.41)	1.40 (0.93)	0.39	15.98	1.04-1.97	0.005***
	DM	43	2.24 (1.58)	1.92 (1.32)	0.70	9.95	1.21-2.53	
	Overall	273	1.84 (1.45)	1.46 (1.07)	0.39	15.98	1.07-2.14	
LDLC	None-DM	224	2.98 (0.96)	2.95 (1.43)	0.49	6.01	2.27-3.70	0.26
	DM	43	2.80 (1.00)	2.69 (1.32)	0.52	5.54	2.14-3.46	
	Overall	267	2.95 (0.97)	2.90 (1.37)	0.49	6.01	2.25-3.62	
HDLC	None-DM	224	1.13 (0.38)	1.10 (0.39)	0.21	2.99	0.95-1.33	0.11
	DM	43	1.05 (0.34)	1.03 (0.29)	0.25	2.35	0.92-1.21	
	Overall	267	1.12 (0.37)	1.09 (0.37)	0.21	2.99	0.94-1.31	
Thyroid								
TSH	None-DM	233	2.14 (1.21)	1.82 (1.49)	0.17	8.44	1.28-2.77	0.92
	DM	43	2.04 (1.11)	1.98 (0.93)	0.20	5.44	1.43-2.36	
	Overall	276	2.12 (1.20)	1.84 (1.39)	0.17	8.44	1.29-2.68	
T4	None-DM	233	58.70 (9.95)	58.97(0)	11.26	114.00	58.97-58.97	0.29
	DM	43	60.42 (6.96)	58.97 (0)	46.50	89.30	58.97-58.97	
	Overall	276	58.97 (9.55)	58.97 (0)	11.26	114.00	58.97-58.97	
T3	None-DM	233	2.86 (0.47)	2.84 (0)	1.37	6.08	2.84-2.84	0.06
	DM	43	2.72 (0.39)	2.84 (0)	1.22	2.84	2.84-2.84	
	Overall	276	2.8 (0.5)	2.84 (0)	1.22	6.08	2.84-2.84	
FT4	None-DM	233	16.49 (2.25)	16.42 (2.76)	10.80	24.14	15.00-17.76	0.54
	DM	43	16.05 (2.43)	16.42 (4.11)	11.10	20.17	13.50-17.61	
	Overall	276	16.42 (2.28)	16.42 (2.95)	10.80	24.14	14.81-17.76	
FT3	None-DM	233	5.09 (0.53)	5.08 (0.72)	3.76	7.32	4.75-5.47	0.16
	DM	43	4.95 (0.53)	4.94 (0.69)	3.60	5.90	4.67-5.36	
	Overall	276	5.07 (0.53)	5.07 (0.77)	3.60	7.32	4.70-5.47	
TPOAb	None-DM	233	45.68 (21.77)	46.58 (8.98)	13.10	270.30	37.60-46.58	0.45
	DM	43	51.44 (42.42)	46.58 (8.40)	12.00	305.20	38.70-47.10	
	Overall	276	46.58 (26.06)	46.58 (8.98)	12.00	305.20	37.60-46.58	
TGAb	None-DM	233	28.92 (19.99)	28.75 (5.25)	10.20	267.60	23.50-28.75	0.47
	DM	43	27.87 (8.75)	28.75 (0)	15.6	76.9	28.75-28.75	
	Overall	276	28.75 (18.68)	28.75 (4.4)	10.2	267.6	24.35-28.75	
Sensitive C-response activation								
HsCRP mg/L (*)	None-DM	228	1.75 (2.99)	0.88 (1.23)	0.05	24.14	0.50-1.73	0.03*
	DM	41	1.85 (1.56)	1.21 (1.93)	0.14	6.83	0.75-2.68	
	Overall	269	1.76 (2.81)	0.92 (1.34)	0.05	24.14	0.50-1.84	
Blood sugar								
GLU (***)	None-DM	229	5.08 (0.86)	4.94(0.94)	3.45	9.94	4.52-5.46	0.00***
	DM	43	7.93 (2.51)	6.98 (3.34)	4.74	14.88	5.98-9.32	
	Overall	272	5.53 (1.64)	5.09 (1.07)	3.45	14.88	4.64-5.71	
HbA1c (***)	None-DM	233	5.66 (0.40)	5.7 (0.6)	4.1	6.4	5.4-6.0	0.00***
	DM	43	7.90 (1.36)	7.6 (1.9)	6.5	11.2	6.7-8.6	
	Overall	276	6.01 (1.04)	5.8 (0.7)	4.1	11.2	5.4-6.1	
Other test								
Hcy	None-DM	233	11.41 (2.78)	11.43 (3.05)	5.40	23.02	9.59-12.64	0.82
	DM	43	11.56 (3.13)	11.10 (3.58)	7.94	23.12	9.40-12.98	
	Overall	276	11.44 (2.83)	11.44 (3.18)	5.40	23.12	9.51-12.69	

Significance is indicated as follows: (*) for p < 0.05, (**) for p < 0.01, and (***) for p < 0.001.

#### 3.1.1 Liver enzymes and kidney function

The comparative analysis revealed no significant differences in liver enzyme levels alanine aminotransferase (ALT) and aspartate aminotransferase (AST) between the DM and non-DM groups. ALT levels averaged 27.77 U/L in the DM group compared to 25.78 U/L in the non-DM group (p = 0.29), while AST levels were 21.28 U/L and 22.01 U/L, respectively (p = 0.41). These findings suggest no apparent hepatic dysfunction associated with T2DM in this cohort.

Similarly, markers of renal function, including blood urea nitrogen (BUN) and serum creatinine, did not differ significantly between the groups. BUN levels averaged 5.61 mmol/L in the DM group and 5.41 mmol/L in the non-DM group (p = 0.15), while creatinine levels were 64.22 µmol/L and 67.30 µmol/L, respectively (p = 0.18). These results indicate preserved renal function across both diabetic and non-diabetic hypertensive patients.

#### 3.1.2 Lipid and inflammatory profiles

A statistically significant difference was observed in triglyceride levels between the two groups, with the DM group demonstrating higher mean levels (2.24 mmol/L) compared to the non-DM group (1.77 mmol/L; *p*=0.005). This finding reinforces the well-established link between elevated triglycerides and increased risk of Type 2 Diabetes Mellitus. In contrast, total cholesterol levels did not significantly differ between groups, suggesting that cholesterol alone may not be a reliable indicator of diabetes risk within hypertensive populations.

Regarding inflammatory markers, the DM group exhibited slightly elevated levels of high-sensitivity C-reactive protein (HsCRP), averaging 1.85 mg/L, compared to 1.75 mg/L in the non-DM group (*p*=0.03). Although the difference was modest, it suggests a low-grade inflammatory state associated with T2DM.

#### 3.1.3 Glycemic markers

As expected, significant differences were observed in glycemic indicators between diabetic and non-diabetic hypertensive patients, clearly reflecting the metabolic impairment associated with Type 2 Diabetes Mellitus. The DM group demonstrated markedly elevated fasting plasma glucose levels, with a mean of 7.93 mmol/L, compared to 5.08 mmol/L in the non-DM group (*p* < 0.001). Similarly, glycated hemoglobin (HbA1c) levels were substantially higher among diabetic individuals, averaging 7.90%, versus 5.66% in the non-DM group (*p* < 0.001).

These statistically significant disparities confirm the presence of poor glycemic control in the diabetic subgroup and align with established diagnostic criteria for T2DM. When viewed in conjunction with elevated triglyceride levels, these findings emphasize the compounded metabolic risk burden among hypertensive individuals with diabetes. This reinforces the need for integrated screening and management strategies targeting both glycemic and lipid abnormalities in this high-risk population.

### 3.2 Analysis of demographic and lifestyle risk factors for type 2 diabetes mellitus

**Figure 1:**
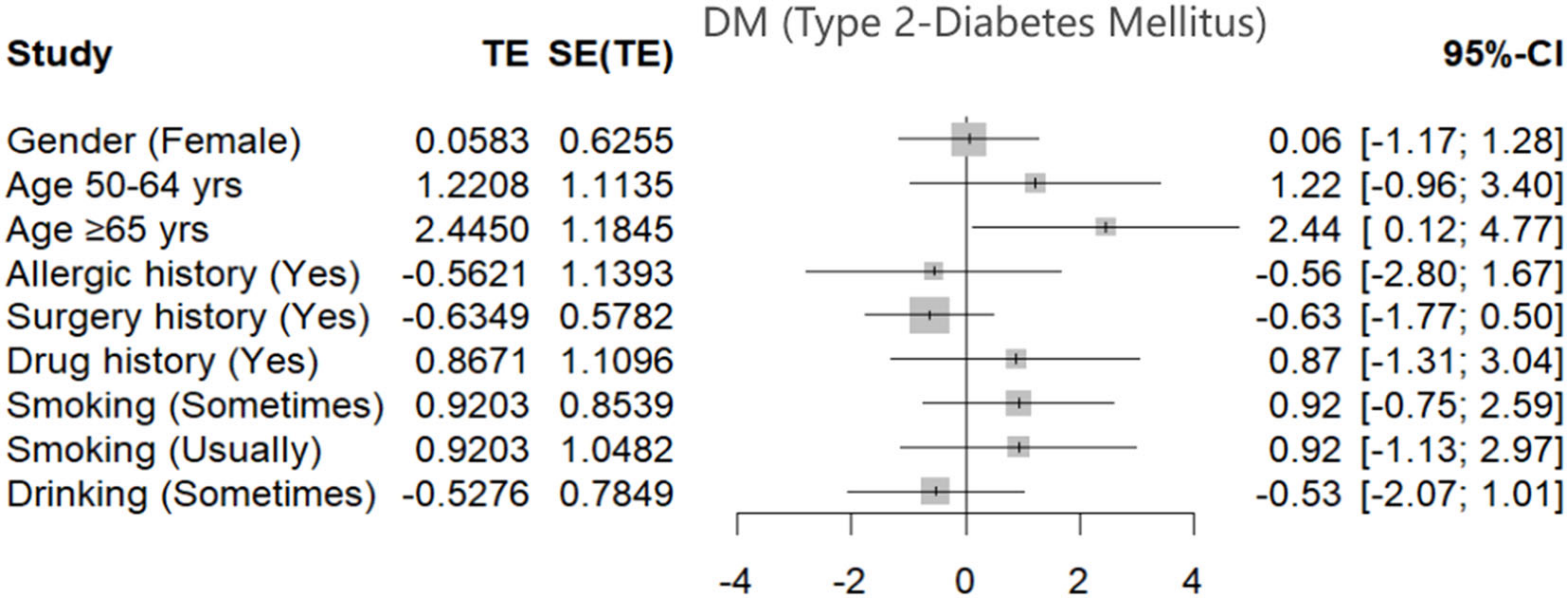
Forest plot shows the estimated effects of demographic and behavioral factors on the risk of Type 2 Diabetes Mellitus (T2DM) among hypertensive patients. Effect sizes (TE) and corresponding 95% confidence intervals (CI) are shown for each variable, including gender, age groups, allergic and surgical history, drug use, and smoking and drinking behaviors. Statistically significant associations were observed for individuals aged ≥65 years, indicating an increased risk of T2DM in this subgroup. Other factors did not demonstrate statistically significant associations within the model.

To evaluate the influence of demographic and lifestyle factors on the risk of Type 2 Diabetes Mellitus (T2DM) among hypertensive patients, a multivariate regression analysis was conducted. The results, presented in **Table 2** and visualized in **Figure 1**, offer a detailed statistical assessment of these associations.

**Table 2:** Demographic characteristics of participants and regression analysis for type-2 diabetes mellitus (DM).

	Total	None-DM	DM	OR value &95%CI	
				Univariate	Multivariate
Gender					
Male	181 (65.58)	154 (66.09)	27 (62.79)	1	1
Female	95 (34.42)	79 (33.91)	16 (37.21)	1.16 (0.59-2.27)	1.33 (0.45-3.89)
Total	276 (100%)	233 (100%)	43 (100%)		
Age groups					
32–49 yrs	62 (22.46)	54 (23.18)	8 (18.60)		
50–64 yrs	143 (51.81)	125 (53.65)	18 (41.86)	0.97 (0.40-2.37)	2.39 (0.57-9.93)
≥65 yrs	71 (25.72)	54 (23.18)	17 (39.53)	2.13 (0.85-5.34)	6.35* (1.26-31.97)
Total	276 (100%)	233 (100%)	43 (100%)		
Allergic history					
No	235 (92.89)	199 (92.99)	36 (92.31)	1	1
Yes	18 (7.11)	15 (7.01)	3 (7.69)	1.11 (0.30-4.01)	0.39 (0.44-3.46)
Total	253 (100%)	214 (100%)	39 (100%)		
Surgery history					
No	173 (63.84)	142 (62.28)	31 (72.09)	1	1
Yes	98 (36.16)	86 (37.72)	12 (27.91)	0.64 (0.31-1.31)	0.57 (0.22-1.48)
Total	271 (100%)	228 (100%)	43 (100%)		
Stroke history					
No	261 (98.86)	219 (98.65)	42 (100.00)	–	–
Yes	3 (1.14)	3 (1.35)	0 (0.00)	–	–
Total	264 (100%)	222 (100%)	42 (100%)		
Drug history					
No	36 (13.38)	33 (14.47)	3 (7.32)	1	
Yes	233 (86.62)	195 (85.53)	38 (92.68)	2.14 (0.63-7.35)	1.43 (0.36-5.67)
Total	269 (100%)	228 (100%)	41 (100%)		
Smoking					
Never	125 (65.45)	106 (66.25)	19 (61.29)	1	1
Sometimes	53 (27.75)	43 (26.88)	10 (32.26)	1.30 (0.56-3.02)	3.92* (1.00-15.35)
Usually	13 (6.81)	11 (6.88)	2 (6.45)	1.01 (0.21-4.94)	1.52 (0.20-11.43)
Total	191 (100%)	160 (100%)	31 (100%)		
Drinking					
Never	113 (59.16)	92 (57.50)	21 (67.74)	1	
Sometimes	75 (39.27)	67 (41.88)	8 (25.81)	0.52 (0.22-1.25)	0.48 (0.13-1.78)
Usually	3 (1.57)	1 (0.63)	2 (6.45)	8.76 (0.76-101.21)	4.01 (0.17-95.80)
Total	191 (100%)	160 (100%)	31 (100%)		

In this table, OR refers to Crude Odds Ratio. The 95% Confidence Interval is denoted as 95% CI. Significance is indicated as follows: (*) for p < 0.05, (**) for p < 0.01, and (***) for p < 0.001.

Age emerged as a significant demographic risk factor. Patients aged 65 years and above exhibited a markedly higher risk of developing T2DM compared to those aged 32–49 years, with an adjusted odds ratio (OR) of 6.35 (95% CI: [1.26, 31.97]). This finding underscores the strong relationship between advancing age and increased diabetes susceptibility in the hypertensive population.

In terms of lifestyle factors, occasional smoking was identified as a notable contributor to elevated diabetes risk. Individuals who reported occasional smoking had an adjusted OR of 3.92 (95% CI: [1.00, 15.35]), indicating a statistically significant increase in T2DM likelihood even with infrequent tobacco exposure.

**Figure 1** presents a forest plot summarizing these findings, displaying the estimated effect sizes and 95% confidence intervals for each demographic and behavioral variable assessed. This graphical representation complements the regression data by visually emphasizing the relative impact and statistical significance of each factor. The plot highlights age and smoking as the most influential variables associated with T2DM risk in this hypertensive cohort, reinforcing the need for early lifestyle intervention and age-specific screening.

### 3.3 Mediation analysis: triglycerides as a mediator between hyperuricemia and type 2 diabetes

To elucidate the mechanistic link between hyperuricemia and Type 2 Diabetes Mellitus (T2DM), a mediation analysis was conducted using generalized structural equation modeling (GSEM). The objective was to assess whether triglyceride levels serve as a mediating factor in this relationship among hypertensive patients. The results, detailed in **Table 3**, indicate that hyperuricemia is significantly associated with elevated triglyceride levels, which in turn are strongly linked to increased diabetes risk. This suggests that the influence of hyperuricemia on T2DM may be exerted indirectly through its effect on lipid metabolism. By quantifying both direct and indirect effects, the GSEM approach provides a robust framework for understanding the intermediary role of triglycerides in the pathophysiology of diabetes development in hypertensive individuals.

**Table 3:** Mediation analysis of hyperuricemia’s effects on type-2 diabetes mellitus (DM) through triglyceride levels.

Variable relationship	Coefficient	Std. error	Z- statistic	P-value	95% confidence interval
Hyperu → TG	0.672	0.271	2.48	0.013*	[0.141, 1.203]
TG → DM	1.294	0.37	3.49	<0.001***	[0.568, 2.020]
Hyperu → DM (Direct)	-0.606	0.371	-1.64	0.102	[-1.333, 0.120]
Hyperu → DM (Indirect via TG)	0.87	0.43	2.02	0.043*	[0.027, 1.712]
Total Effect Hyperu → DM	0.263	0.534	0.49	0.622	[-0.783, 1.310]

*** indicates p-value <0.001; * indicates p-value<0.05.

#### 3.3.1 Direct and indirect effects

**Figure 2:**
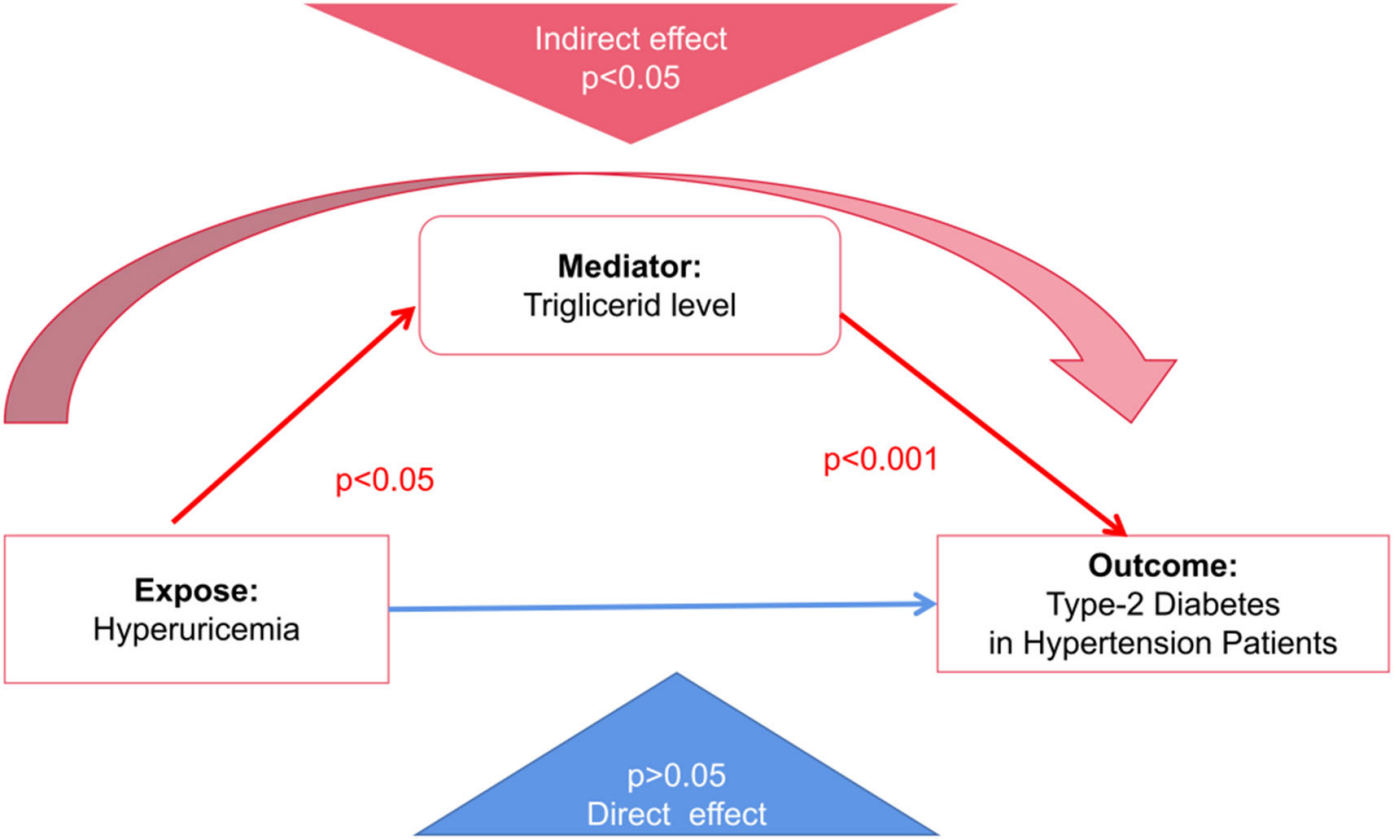
Mediation model illustrating the indirect effect of hyperuricemia on Type 2 Diabetes Mellitus (T2DM) in hypertensive patients through triglyceride levels. The red arrows represent statistically significant indirect pathways (*p* < 0.05 for hyperuricemia → triglycerides; *p* < 0.001 for triglycerides → T2DM). The blue arrow indicates a non-significant direct effect of hyperuricemia on T2DM (*p* > 0.05). This model supports the role of triglyceride levels as a significant mediator in the metabolic pathway linking hyperuricemia to diabetes risk.

The mediation analysis revealed that hyperuricemia, when considered independently, did not exhibit a statistically significant direct effect on the risk of developing Type 2 Diabetes Mellitus (coefficient = -0.606, *p*=0.10). However, a significant indirect effect was observed through elevated triglyceride levels (coefficient = 0.87, *p*=0.04). This finding underscores the critical mediating role of triglycerides, suggesting that the impact of hyperuricemia on diabetes risk is primarily exerted via its influence on lipid metabolism rather than through a direct pathway. The significant indirect association highlights triglycerides as a key metabolic intermediary in the progression from hyperuricemia to T2DM within hypertensive populations (**Figure 2**).

#### 3.3.2 Statistical significance and confidence intervals

The mediation model demonstrated a statistically significant positive association between serum uric acid levels and triglyceride concentrations, with a path coefficient of 0.672 (*p*=0.013). This finding supports the hypothesis that hyperuricemia contributes to dysregulated lipid metabolism. Importantly, elevated triglyceride levels were also significantly associated with an increased risk of Type 2 Diabetes Mellitus, with a path coefficient of 1.294 (*p* < 0.001). These results reinforce the role of triglycerides as a metabolic bridge linking hyperuricemia to diabetes risk. From a clinical perspective, they suggest that interventions targeting triglyceride reduction may offer a promising strategy for mitigating diabetes risk in individuals with elevated uric acid levels, particularly within hypertensive populations.

#### 3.3.3 Total effect analysis

Although the total effect of hyperuricemia on Type 2 Diabetes Mellitus was not statistically significant (coefficient = 0.26, *p*=0.62), the indirect effect mediated through triglyceride levels accounted for a substantial proportion of the overall estimated effect. Although the calculated proportion exceeded 100% (approximately 330%), this may reflect suppression effects or statistical scaling in non-standardized coefficients, which are known to occur in mediation models, especially with small effect sizes or imbalanced sample structures. These estimates should be interpreted cautiously and do not imply a mechanistically exaggerated effect. This disproportionate mediation underscores the dominant role of triglycerides in linking hyperuricemia to diabetes risk.

These findings further validate the critical intermediary function of triglycerides in the metabolic pathway between elevated uric acid levels and the onset of diabetes. From a clinical standpoint, this analysis highlights the importance of integrating triglyceride management into diabetes prevention strategies, especially for hypertensive patients with hyperuricemia. The results also call for future research to unravel the underlying biological mechanisms and to inform the development of targeted interventions that disrupt this pathogenic cascade.

### 3.4 Implications of BMI on metabolic dysfunctions in hypertensive diabetic patients

Building on the findings from Section 3.3, this section explores the influence of body mass index (BMI) on critical metabolic parameters—specifically hyperuricemia, triglyceride levels, and the prevalence of Type 2 Diabetes Mellitus (T2DM)—within the hypertensive diabetic population.

The analysis reveals a clear pattern: as BMI increases, so does the prevalence of both hyperuricemia and elevated triglyceride levels. Patients in the highest BMI category (≥27 kg/m²) exhibited the greatest incidence of these metabolic disturbances, suggesting a strong positive association between excess adiposity and metabolic dysfunction. These findings reinforce the role of obesity as a central contributor to metabolic derangements, particularly in individuals already burdened by hypertension and diabetes. Interestingly, while BMI was significantly associated with elevated uric acid and triglyceride levels, its direct relationship with T2DM was not statistically significant. This may be due to BMI’s limitation in capturing fat distribution, particularly visceral and hepatic fat, which are more strongly implicated in metabolic dysfunction. Central obesity and fatty liver rather than generalized obesity are increasingly recognized as critical contributors to insulin resistance and diabetes development. Thus, BMI may exert its influence on diabetes risk indirectly, by reflecting an underlying increase in ectopic fat that drives dyslipidemia and hyperuricemia.

Interestingly, while BMI was significantly associated with elevated uric acid and triglyceride levels, its direct relationship with T2DM was not statistically significant. This suggests that BMI may exert its influence on diabetes risk indirectly—potentially through intermediary pathways such as dyslipidemia and hyperuricemia, rather than serving as a direct causal factor.

Overall, these observations emphasize the multifaceted role of BMI in metabolic health and highlight the necessity of comprehensive weight management strategies. Addressing obesity could indirectly modulate both uric acid and lipid profiles, thereby reducing the overall metabolic burden in hypertensive diabetic patients.

#### 3.4.1 Association of BMI categories with hyperuricemia and elevated triglyceride levels

As summarized in **Table 4**, a statistically significant positive correlation was observed between increasing BMI categories and the prevalence of both hyperuricemia and elevated triglyceride levels two key markers of metabolic dysfunction. The data indicate that with each successive BMI category, the likelihood of hyperuricemia increases by approximately 50%, underscoring a substantial metabolic burden. This trend is particularly concerning given hyperuricemia’s established role in exacerbating renal and cardiovascular complications.

**Table 4:** Effects of BMI categories on hyperuricemia and triglyceride levels.

Outcome variable	Predictor	Coefficient	Std. error	Z-statistic	P-value	95% confidence interval
Hyperuricemia	BMI	0.484	0.138	3.51	<0.001 ***	[0.214, 0.754]
Triglyceride Levels	BMI	0.436	0.120	3.64	<0.001***	[0.201, 0.670]

*** indicates p-value <0.001.

Similarly, triglyceride levels demonstrated a marked rise across higher BMI categories. This elevation is especially relevant in the context of Type 2 Diabetes Mellitus, as hypertriglyceridemia not only complicates glycemic control but also significantly heightens cardiovascular risk. These findings highlight the compounded metabolic challenges faced by obese hypertensive patients and reinforce the importance of early, targeted interventions aimed at weight reduction and lipid regulation to mitigate downstream health consequences.

#### 3.4.2 Categorical analysis of BMI and its impact on type-2 diabetes mellitus

**Table 5** presents a categorical analysis of BMI and its relationship with key metabolic conditions, offering insight into how increasing BMI influences the prevalence of hyperuricemia, elevated triglyceride levels, and Type 2 Diabetes Mellitus (T2DM). The data reveal a clear upward trend in metabolic risk with increasing adiposity. The most pronounced findings emerge from the highest BMI category (≥27 kg/m²), where 60.26% of individuals exhibited hyperuricemia and 38.46% had elevated triglyceride levels. These prevalence rates are substantially greater than those observed in lower BMI categories, emphasizing the heightened metabolic risk associated with obesity in hypertensive patients. These trends underscore the urgent need for targeted weight management and metabolic monitoring in this subgroup.

**Table 5:** Categorical distribution of metabolic conditions across BMI categories.

BMI category	% with hyperuricemia	% with elevated triglycerides (≧2.0 mmol/l triglyceride level)	% with type-2 diabetes
<22	25.00%	17.86%	14.29%
22-24	36.67%	16.67%	10.00%
24-27	41.82%	27.27%	15.45%
≧27	60.26%	38.46%	20.51%
P-value	0.003**	0.032*	0.407

** indicates p-value <0.01; *indicates p-value <0.05.

However, despite the increasing prevalence of hyperuricemia and dyslipidemia with higher BMI, the direct association between BMI and the prevalence of T2DM did not reach statistical significance in this model. This suggests that BMI may influence diabetes risk indirectly potentially through intermediary mechanisms such as lipid and uric acid dysregulation or that other unmeasured confounding factors (e.g., genetic predisposition, dietary habits, or physical activity levels) may be playing a role.

These findings highlight the complexity of metabolic interactions and the need for multifactorial approaches in diabetes prevention, particularly within obese hypertensive populations.

## 4 Discussion

This study elucidates the complex interplay between metabolic, demographic, and lifestyle factors and their combined influence on the risk of developing Type 2 Diabetes Mellitus (T2DM) in hypertensive patients. The comprehensive analysis provides meaningful insights into the underlying mechanisms of disease progression and highlights potential targets for preventive strategies in this high-risk population.

Recent evidence suggests an emerging association between hyperuricemia (HUA), hypertension (HTN), and T2DM (23). Consistent with this, our mediation analysis explored both direct and indirect pathways linking these conditions. Although the total effect of hyperuricemia on diabetes risk was not statistically significant (coefficient = 0.26, *p*=0.62), the indirect effect through elevated triglyceride levels was substantial and statistically significant. Our findings indicate inconsistent mediation/suppression: TG conveys a positive, statistically significant indirect association between HUA and T2DM, whereas the direct association was negative and non-significant. Given this sign discordance and a near-zero total effect, we do not present “percent mediated” and instead emphasize the magnitude and uncertainty of the indirect path.

This finding underscores the importance of triglycerides as a pivotal metabolic factor connecting hyperuricemia to diabetes. While hyperuricemia alone may not significantly elevate the risk of diabetes in hypertensive individuals, its influence appears to be exerted indirectly through dysregulation of lipid metabolism. These results highlight the potential benefit of incorporating triglyceride management into comprehensive diabetes prevention strategies, particularly for hypertensive patients with elevated serum uric acid levels. However, our findings must be interpreted with caution given the cross-sectional study design, which limits causal inference. While GSEM allows estimation of direct and indirect pathways, it does not establish temporality. The observed suppression effects between direct and indirect paths further highlight the complexity of this relationship. Additionally, potential residual confounding may persist despite adjustment for key covariates, including age, sex, BMI, smoking, alcohol use, renal function, and relevant medications. Specifically, the combination of a negative direct effect (-0.61) and a positive indirect effect (0.87) suggests that the mediator (triglycerides) absorbs or masks part of the true effect a known issue in structural models. Additionally, unmeasured confounding factors, such as the proposed antioxidant properties of uric acid, could influence this pathway. The finding that BMI correlates with triglycerides and uric acid but not directly with diabetes may also reflect the inadequacy of BMI as a measure of visceral fat or liver lipid burden key players in insulin resistance. Future research should employ bootstrapped confidence intervals to verify the robustness of the indirect effect and further investigate these mechanistic complexities.

Notably, our findings are consistent with the “lipid overflow” hypothesis described by Taylor and colleagues, which proposes that ectopic fat accumulation in the liver and pancreas driven by excessive triglyceride spillover is a fundamental cause of Type 2 Diabetes Mellitus. According to this view, hypertriglyceridemia originates from fatty liver outflow, which also impacts uric acid dynamics. While our study did not directly assess hepatic fat, the observed mediation of hyperuricemia’s effect on diabetes via triglycerides supports this mechanistic pathway. Importantly, these insights suggest that triglyceride-lowering interventions rather than uric acid reduction alone may offer more effective preventive strategies for T2DM in metabolically vulnerable populations.

It is also important to distinguish between BMI and central obesity. While BMI was not directly associated with diabetes risk in our model, this does not preclude the pathogenic role of visceral adiposity. Fat accumulation in the liver (i.e., non-alcoholic fatty liver disease) is increasingly viewed as a central mechanism driving insulin resistance and hypertriglyceridemia. Triglyceride overspill from fatty liver into the circulation may also explain the elevated triglyceride levels observed in hypertensive diabetic patients. In this context, hyperuricemia may be a secondary marker of metabolic imbalance rather than a direct causal factor. Therefore, targeting hepatic triglyceride production and promoting visceral fat reduction could be more effective strategies for diabetes prevention than uric acid-lowering therapies alone.

Hyperuricemia (HUA) is increasingly recognized as an independent risk factor for the development of Type 2 Diabetes Mellitus (T2DM). Systematic reviews and meta-analyses have consistently demonstrated that each 1 mg/dL increase in serum uric acid (UA) levels is associated with approximately a 6% higher risk of T2DM, with a positive nonlinear relationship observed between UA levels and both T2DM and impaired fasting glucose (IFG) incidence (24, 25). The risk conferred by HUA appears to differ by comorbidity and sex, with studies indicating a higher diabetes risk among patients with gout compared to those with osteoarthritis, and distinct risk profiles observed between men and women likely influenced by hormonal differences in UA excretion (26–28).

Experimental studies provide mechanistic support for these observations, showing that elevated UA levels exacerbate inflammatory responses and oxidative stress, which in turn impair both insulin sensitivity and pancreatic β-cell function (29–31). These effects contribute directly to the pathogenesis of T2DM. Additionally, pharmacological interventions such as allopurinol have demonstrated efficacy in improving insulin resistance and glycemic control in patients with T2DM and coexisting HUA, suggesting that urate-lowering therapy may offer metabolic benefits beyond gout management (32, 33).

Together, these findings reinforce the critical role of uric acid in glucose metabolism and support the clinical relevance of targeting UA levels as part of a broader strategy for the prevention and management of T2DM, particularly in hypertensive populations already at elevated metabolic risk.

In addition to its association with diabetes, hyperuricemia (HUA) has been consistently linked to an elevated risk of hypertension (HTN). Epidemiological studies show that for every 1 mg/dL increase in serum uric acid (UA) levels, there is an associated 13% rise in the incidence of HTN, with UA levels exceeding 6.5 mg/dL correlating with a 25% increased risk even among otherwise healthy individuals (34, 35). This relationship appears to be partly mediated by renal function, as UA levels have been associated with renal biomarkers such as serum creatinine and estimated glomerular filtration rate (eGFR), suggesting that hyperuricemia may contribute to blood pressure elevation through renal impairment (34, 36).

Experimental studies in animal models further support these findings. Elevated UA levels have been shown to induce both hypertension and renal pathologies in rats, offering mechanistic insights into how hyperuricemia may influence vascular resistance and kidney function (37, 38). Clinical trials lend additional weight to these observations, demonstrating that urate-lowering therapies, particularly allopurinol, significantly reduce both systolic and diastolic blood pressure in patients with coexisting HUA and HTN (39–41). These cumulative findings reinforce the strong link between hyperuricemia and hypertension and highlight the potential therapeutic benefits of UA management in controlling blood pressure, especially in metabolically vulnerable populations.

In interpreting our findings, it is essential to clarify the study population structure: all participants in our cohort were diagnosed with hypertension at baseline. These individuals were then categorized into two subgroups based on the presence or absence of Type 2 Diabetes Mellitus (T2DM): one group comprised patients with both hypertension and diabetes, while the other included those with hypertension but without diabetes. This classification is crucial, as it directly influences the interpretation of our results. For clarity and brevity within the text, we occasionally refer to individuals with both conditions simply as “diabetic patients,” though it should be understood that all study participants had hypertension, and the diabetes status served as the distinguishing variable for subgroup analysis.

Our regression analysis revealed that hyperuricemia alone did not significantly increase the risk of diabetes among hypertensive patients (coefficient = -0.606, *p*=0.10). However, the mediation model demonstrated a significant indirect effect through elevated triglyceride levels (coefficient = 0.87, *p*=0.04), suggesting that the effect of hyperuricemia on diabetes risk becomes evident only when triglyceride levels are considered as a mediating factor. Without this mediation analysis, the influence of hyperuricemia on diabetes risk in hypertensive individuals might appear negligible.

Demographic analysis further highlighted age as a significant risk factor for diabetes. Participants aged 65 years and older exhibited a markedly higher risk of developing T2DM, with a multivariate odds ratio (OR) of 6.35 and a 95% confidence interval (CI) of [1.26, 31.97] (*p* < 0.05). While the role of gender in diabetes risk was not directly assessed in terms of uric acid stratification, age was prioritized in our model as a non-modifiable but influential factor. Our primary analytical focus remained on modifiable lifestyle risks.

One notable finding was the significant association between occasional smoking and increased risk of diabetes. Individuals who reported occasional tobacco use had a multivariate OR of 3.92 (95% CI: [1.00, 15.35]; *p* < 0.05), indicating that even infrequent smoking contributes meaningfully to diabetes susceptibility among hypertensive patients. Interestingly, this association was not observed in individuals who reported frequent or not smoking, suggesting that the metabolic effects of intermittent tobacco exposure may follow a unique risk pattern. Conversely, alcohol consumption, whether frequent, occasional, or abstinent, did not demonstrate a statistically significant impact on diabetes risk. Although the multivariate OR for frequent alcohol use was 4.01 (95% CI: [0.17, 95.80]), the wide confidence interval and *p* > 0.05 indicate a lack of significant association in our cohort.

These observations naturally prompt consideration of the broader relationship between hypertension and T2DM a complex, bidirectional dynamic shaped by overlapping metabolic, inflammatory, and lifestyle-related mechanisms.

Hypertension (HTN) and Type 2 Diabetes Mellitus (T2DM) are well-documented comorbidities that frequently coexist and mutually exacerbate cardiovascular and metabolic risk. Mendelian randomization (MR) analyses have provided compelling evidence suggesting that T2DM may causally influence the development of hypertension, whereas the reverse relationship appears less definitive (17). This directional influence underscores the need to further investigate the molecular pathways connecting these conditions, with hyperinsulinemia emerging as a potential therapeutic target.

Globally, both HTN and T2DM are major contributors to morbidity and mortality, ranking among the most critical modifiable cardiovascular risk factors (18–20). Their frequent coexistence amplifies the risk of adverse outcomes, including cardiovascular events, renal dysfunction, and chronic organ failure. Evidence supports a bidirectional relationship, wherein each condition potentiates the progression of the other (42, 43). At the pathophysiological level, shared mechanisms such as insulin resistance, chronic hyperinsulinemia, systemic inflammation, and oxidative stress underlie the mutual reinforcement of T2DM and HTN (44, 45).

Recent MR studies have further reinforced the role of T2DM as a driver of hypertension, suggesting that effective management of hyperglycemia may also reduce blood pressure and delay the onset of hypertension (17). However, the inverse whether controlling hypertension can prevent or mitigate diabetes remains less clear and warrants further investigation.

It is important to consider that all individuals in our study were managing hypertension with medication, which may have influenced both glycemic control and the progression of T2DM. This clinical context is crucial when interpreting our findings, as antihypertensive therapies themselves can modulate metabolic outcomes, potentially confounding the observed associations between hyperuricemia, triglycerides, and diabetes risk.

Our findings highlight triglycerides as a significant mediator in the relationship between hyperuricemia and Type 2 Diabetes Mellitus (T2DM). The mediation analysis demonstrated a strong positive association between elevated serum uric acid levels and increased triglyceride concentrations (coefficient = 0.672, *p*=0.013), suggesting that dyslipidemia may serve as a critical link in this metabolic cascade. Moreover, high triglyceride levels were found to significantly increase the likelihood of diabetes (coefficient = 1.294, *p* < 0.001), reinforcing the importance of lipid regulation in diabetes risk management.

These findings emphasize the clinical potential of targeting triglycerides to reduce T2DM risk, particularly among individuals with hyperuricemia. From a mechanistic standpoint, triglyceride accumulation reflects hepatic lipid overflow—a process tightly linked to hyperuricemia through fructose metabolism. Excessive fructose intake undergoes rapid phosphorylation by fructokinase, depleting intracellular ATP and generating AMP, which is subsequently degraded to uric acid. Concurrently, fructose bypasses the key glycolytic control step at phosphofructokinase, driving unregulated *de novo* lipogenesis in hepatocytes. This dual pathway simultaneously elevates uric acid and triglyceride synthesis, establishing a biochemical crosstalk wherein hyperuricemia promotes lipogenesis and hypertriglyceridemia exacerbates uric acid production via oxidative stress and insulin resistance. In the context of our mediation analysis, this synergy provides a plausible biological explanation for the observed suppression effect—whereby the positive indirect pathway through triglycerides is counterbalanced by a negative direct pathway from uric acid to diabetes. Such antagonistic effects within the same causal framework underscore the metabolic complexity of fructose-driven hepatic dysregulation. This biochemical synergy between uric acid and lipid production provides a plausible pathway through which hyperuricemia indirectly promotes diabetes risk via triglyceride elevation. The observed disconnect between BMI and diabetes risk also warrants deeper interpretation. Rather than dismissing this as a paradox, it more likely reflects the limited ability of BMI to capture central obesity and visceral fat burden—both of which are stronger predictors of insulin resistance than total body mass. Our results suggest that body fat distribution, particularly hepatic and visceral adiposity, may mediate the observed metabolic disturbances more than BMI alone.

In terms of intervention, the call for “lipid regulation” must be specific. Lifestyle approaches including dietary fructose restriction, increased physical activity, and weight reduction remain foundational. Pharmacologic options may include fibrates, omega-3 fatty acids, or newer agents like pemafibrate, which have demonstrated triglyceride-lowering and potential insulin-sensitizing effects. Importantly, hypertensive individuals often receive medications such as thiazide diuretics or beta-blockers, which themselves influence glucose and lipid metabolism. This population’s unique pathophysiological profile—combining vascular dysfunction, metabolic dysregulation, and medication-induced shifts justifies its selection as a high-risk, clinically actionable subgroup for targeted diabetes prevention strategies.

Hyperuricemia itself is a growing global concern, affecting approximately 21% of adults in the United States (46), 13.3% in China (47), and 25.8% in Japan (48). Despite its high prevalence and its known implications for metabolic and cardiovascular health, the underlying pathogenic mechanisms remain incompletely understood. Triglycerides, as the dominant form of lipid storage in adipose tissue, have been increasingly implicated in the pathogenesis of hyperuricemia. Multiple studies have reported a positive correlation between triglyceride levels and the risk of developing hyperuricemia (49–51). However, much of the existing research has been limited by a narrow demographic focus particularly on male populations and by insufficient adjustment for potential confounding variables (52, 53). These gaps highlight the need for broader, more inclusive research to fully elucidate the lipid-uric acid axis and its role in metabolic disorders.

The role of triglycerides in the development of Type 2 Diabetes Mellitus (T2DM) is of growing clinical concern, particularly given the rising global burden of diabetes. In China, diabetes affects approximately 11.6% of the adult population, with projections indicating a substantial increase in prevalence across developing countries by 2030 (54, 55). T2DM significantly elevates the risk for both cardiovascular disease and chronic kidney disease, contributing to considerable morbidity and mortality worldwide (56, 57).

Triglycerides (TGs), the primary lipids stored in human adipose tissue, have emerged as key metabolic indicators in this context. According to the National Cholesterol Education Program (NCEP) Adult Treatment Panel III guidelines (2001), TG levels are categorized as desirable (<1.7 mmol/L), borderline-high (1.7–2.25 mmol/L), high (2.26–5.6 mmol/L), and very high (≥5.6 mmol/L). Numerous studies have reported a linear correlation between rising TG levels and increased T2DM risk, reinforcing triglycerides as an essential biomarker for early diabetes prediction (58–61).

Despite the strength of this association, further longitudinal and multiethnic research is warranted to validate these findings, particularly with adjustments for confounding variables such as dietary patterns, physical activity, and genetic predispositions. Such studies will be crucial to confirming triglycerides’ independent predictive value and to guiding more effective risk stratification and intervention strategies.

Hypertension remains a pressing global health challenge, particularly in low- and middle-income countries, where it contributes substantially to morbidity and mortality (62, 63). Early identification of individuals at high risk and timely intervention are essential strategies to curb the incidence of hypertension and its associated cardiovascular complications (64). While traditional methods for assessing insulin resistance such as the hyperinsulinemic–euglycemic clamp are considered gold standards, they are impractical for routine clinical use due to their complexity and cost (65).

In this context, the triglyceride-glucose (TyG) index has emerged as a practical, non-insulin-based surrogate marker for insulin resistance. It has shown strong correlations with increased risks of both T2DM and, potentially, hypertension, although existing studies have yielded mixed results (66–70). Notably, a large-scale Japanese cohort study found that both fasting and non-fasting serum triglyceride levels were independently associated with the onset of hypertension, even after adjusting for a wide range of lifestyle and metabolic confounders (7). These findings are supported by additional research conducted across diverse populations, which consistently demonstrate a positive association between elevated triglyceride levels and hypertension risk (7–13, 16). The consistency of these results across different age groups, sexes, and geographic regions underscores the clinical relevance of triglycerides as a modifiable risk factor in hypertension prevention strategies.

The relationship between serum triglycerides, visceral fat accumulation, and insulin resistance suggests that triglycerides may function as more than just a metabolic marker they may serve as a direct risk factor for the development of hypertension (71, 72). This hypothesis is further supported by interventional studies demonstrating that triglyceride-lowering treatments can lead to reductions in blood pressure, thereby implying a potential causal link between triglyceride levels and hypertension risk (73–75). These findings reinforce the importance of lipid regulation, not only in the context of diabetes prevention but also as a viable target in hypertension management strategies.

This study underscores the importance of integrating comprehensive metabolic risk assessments into routine clinical care for hypertensive patients, particularly those at risk for Type 2 Diabetes Mellitus (T2DM). In addition to conventional biomarkers such as blood glucose and HbA1c, the inclusion of triglyceride measurements enhances the early detection of metabolic dysfunction and improves risk stratification (76, 77). These combined markers offer a more complete clinical picture, facilitating timely intervention and potentially improving long-term outcomes.

Beyond clinical assessment, the findings emphasize the effectiveness of lifestyle interventions in reducing diabetes risk, especially among older adults and individuals engaging in modifiable high-risk behaviors. Community-based lifestyle modification programs including those focused on dietary changes, physical activity, and smoking cessation have demonstrated significant success in lowering the incidence of T2DM and improving metabolic health at the population level (78–80). These results highlight the value of targeted public health strategies aimed at behavioral risk reduction in vulnerable populations.

Together, the dual approach of evidence-based clinical management and scalable public health interventions aligns with current medical guidelines and supports the broader goals of national and international public health frameworks. By addressing both individual-level and systemic risk factors, these integrative strategies are essential for reducing the burden of diabetes in hypertensive populations and improving overall public health outcomes.

This study provides important insights into the mediating role of triglycerides in the association between hyperuricemia and diabetes risk among hypertensive individuals. Using advanced statistical modeling, including generalized structural equation modeling (GSEM), we demonstrate that triglyceride levels significantly mediate this relationship, even though the direct effect of hyperuricemia on diabetes did not reach statistical significance. These findings contribute to the growing body of evidence highlighting the central role of lipid metabolism in the pathogenesis of Type 2 Diabetes Mellitus.

A major strength of the study lies in the use of a robust clinical dataset and comprehensive metabolic profiling, which allowed for a nuanced analysis of direct and indirect effects. However, several limitations must be acknowledged. First, the study design was cross-sectional, limiting our ability to infer causal relationships or temporal sequences between hyperuricemia, triglycerides, and diabetes onset. Second, while the overall cohort size was moderate, the number of participants with Type 2 Diabetes Mellitus (T2DM) was relatively small, which may affect the robustness of subgroup analyses and limit the generalizability of our findings. Larger-scale studies are warranted to confirm these observations and strengthen statistical power.

To build upon these findings, future research should prioritize prospective, longitudinal studies that can more definitively establish causal links and clarify the temporal sequence of metabolic changes. In-depth exploration of the biochemical mechanisms underlying the triglyceride-hyperuricemia-diabetes axis is also warranted. Moreover, randomized controlled trials examining lifestyle or pharmacological interventions targeting triglyceride levels could provide practical insights into effective prevention strategies for diabetes in hypertensive populations. Such studies have the potential to refine clinical guidelines and improve outcomes through more personalized, metabolically informed approaches to care.

Another important limitation involves potential confounding by medication use, which was not fully adjusted in our analyses. Antihypertensive, lipid-lowering, or glucose-lowering agents could influence uric acid or triglyceride levels, thus affecting mediation estimates. Furthermore, the use of GSEM in a cross-sectional framework precludes definitive causal interpretation. While mediation models imply directional pathways, true mediation requires longitudinal or experimental data. Our findings should thus be interpreted as exploratory, warranting confirmation through prospective or interventional studies with more robust control of confounding variables.

## 5 Conclusion

This study reinforces the pivotal role of triglycerides as a mediator linking hyperuricemia and Type 2 Diabetes Mellitus (T2DM) among hypertensive individuals. Notably, our findings suggest that hyperuricemia may not directly increase diabetes risk, but exerts its effect indirectly via elevated triglyceride levels. This aligns with emerging mechanistic models, including studies by Taylor and others, which propose that triglyceride overspill from ectopic fat depots particularly the liver and pancreas is central to the development of T2DM. These findings support the hypothesis that central obesity and hepatic steatosis, rather than general BMI, are key drivers of metabolic dysfunction. From a clinical standpoint, our findings suggest that in hypertensive individuals at high risk for diabetes particularly those with prediabetes priority should be given to triglyceride-lowering interventions before urate-lowering strategies. Elevated triglycerides appear to be a more proximal driver of diabetes risk, mediating much of hyperuricemia’s effect through hepatic lipid overflow and insulin resistance. Lifestyle measures such as dietary fructose restriction, increased physical activity, and weight reduction should be implemented as first-line approaches. Pharmacologic options including fibrates, omega-3 fatty acids, and selective PPARα modulators may be considered when lifestyle modification alone is insufficient. Urate-lowering therapy may still be warranted in patients with symptomatic hyperuricemia or gout, but in the context of diabetes prevention, it is likely to be most effective when combined with triglyceride-targeted interventions. Future longitudinal and interventional studies should evaluate optimal sequencing and synergy between these treatment strategies to maximize metabolic benefit in hypertensive prediabetic populations.

## Data Availability

The original contributions presented in the study are included in the article/supplementary material. Further inquiries can be directed to the corresponding author.
